# Large species within carnivora are large carnivores

**DOI:** 10.1098/rsos.181228

**Published:** 2018-09-12

**Authors:** Christopher Wolf, Matthew G. Betts, Taal Levi, Thomas M. Newsome, William J. Ripple

**Affiliations:** 1 Global Trophic Cascades Program, Department of Forest Ecosystems and Society, Oregon State University, Corvallis, OR 97331, USA; 2 Department of Fisheries and Wildlife, Oregon State University, Corvallis, OR 97331, USA; 3 School of Life and Environmental Sciences, The University of Sydney, New South Wales 2006, Australia

## 1. Introduction

Wolf & Ripple [1] is a global study that explored the potential for large carnivore reintroductions, identifying protected areas and other regions with low human footprint that warrant further investigation as possible locations for rewilding. In his comment, Miranda [2] raised a number of points related to Wolf & Ripple [1]. His primary points are that Wolf and Ripple (i) used terminology (carnivore, apex predator, and guild) incorrectly, (ii) selected an inappropriate group of species to study and (iii) listed some unsuitable sites for large carnivore reintroductions. However, each of these criticisms is fundamentally flawed and only acts to hinder conservation efforts.

## 2. Scientific terminology

Miranda stated that the species selected by Wolf and Ripple ‘were neither necessarily apex predators nor carnivores, but a subset of the mammalian order Carnivora, […]'. However, Wolf and Ripple did not claim that the species being studied are apex predators. In fact, the term ‘apex predator' does not appear anywhere in the main text or supplement of Wolf and Ripple. The species selected included 25 large carnivore species with a body mass greater than or equal to 15 kg that have relatively accurate historic and current range maps. The scope and purpose of Wolf and Ripple was to identify potential reintroduction sites for large-bodied members of order Carnivora without regard for whether each individual species was an apex predator, which is in itself context dependent. For example, coyotes (*Canis latrans*) may be considered mesopredators when wolves are present, but apex predators when wolves are absent [3]. Had Wolf and Ripple focused on potential reintroductions of a different taxonomic order, such as Primates, would Miranda then critique that the paper did not focus enough on other groups of vertebrates?

Miranda is incorrect in his claim that the species selected are not carnivores because many do not have strictly carnivorous diets. The term ‘carnivore' can properly refer to either the diet of an organism or the taxonomic order ‘Carnivora'. Wolf and Ripple specified that the species in their analysis are ‘carnivores (members of the mammalian order Carnivora)', making it clear that the term was intended in this context rather than as a reference to hyper-carnivorous animals in general. Although the appropriateness of referring to members of order Carnivora as ‘carnivores' seems self-evident, we refer Miranda to the Encyclopedia of Life, which lists ‘carnivores' as the preferred (and only) English common name for the mammal order Carnivora [4]. The Encyclopedia of Life lists this common name as ‘Trusted' and cites seven sources for it, including the integrated taxonomic information system (ITIS) and the National Center for Biotechnology Information (NCBI) Taxonomy Database.

According to Miranda, Wolf and Ripple ‘confuse guilds (a group of species that exploit similar resources) with taxocenosis (a group of sympatric species sharing a common phylogenetic clade)'. However, Wolf and Ripple's use of the term guild is common in the carnivore literature. A Web of Science Topic search (title, abstract and keywords) spanning 1965–2018 for ‘carnivore guild' returned 88 results. This search did not include the text of papers, making the total an underestimate of the term's frequency of occurrence. Many of the results were papers that grouped omnivores such as the brown hyena (*Hyaena brunnea*), striped hyena (*Hyaena hyaena*) and most bear species (*Ursidae*) together with obligate meat-eating carnivores into a single large carnivore guild (e.g. [5–9]). For example, here are some quotes on large carnivore guilds from other references that are consistent with Wolf and Ripple's use of the term:

‘the large carnivore guild (i.e. brown hyaena *Hyaena hyaena*, spotted hyaena *Crocuta crocutta*, cheetah *Acinonyx jubatus*, leopard *Panthera pardus*, lion *Panthea leo* and African wild dog *Lycaon pictus*)' [10]

‘[…] wolves are members of a guild that includes other large carnivores, such as bears and cougars.' [11]

‘members of the large carnivore guild, such as ursids (Carnivora, Ursidae), felids (Carnivora, Felidae) and canids (Carnivora, Canidae)' [12]

So, Wolf and Ripple's usage of the term ‘carnivore guild' is in keeping with common use of this term in the scientific literature.

## 3. Species analysed

According to Miranda, ‘Wolf and Ripple ignore many of the Earth's most quintessential predators'. He claims that this is an example of ‘Taxonomic chauvinism'. This claim is unreasonable given that the terrestrial large carnivores considered by Wolf and Ripple have many important similarities that justify them being considered as a group. They tend to be charismatic, flagship species whose conservation can benefit other taxa; have significant overlap in terms of threats faced (e.g. conflict with livestock); and have high quality data available, including historical range maps, which are critical to a study of reintroduction possibilities [13,14].

Miranda failed to provide any evidence for his assertion that trophic cascade ecology has ‘conspicuously focused on the Carnivora mammal taxocenosis'. A substantial portion of published trophic cascades articles relate to freshwater ecosystems, which were the primary focus of early research [15,16]. Between 1988 and 2015, the number of articles on aquatic trophic cascades (1087 articles in total: 477 on freshwater trophic cascades and 610 on marine trophic cascades) was 50% greater than the number of articles on terrestrial trophic cascades (723) [16]. Moreover, many terrestrial-focused studies involved predators outside Carnivora such as arachnids (Arachnida), insects (Insecta), reptiles (Reptilia) and raptors [17–21].

## 4. Specific reserves' suitability

Miranda criticized the suitability of specific reserves listed in Wolf and Ripple. These criticisms are peculiar given that the page-long limitations section in Wolf and Ripple states: ‘All results that we present need to be more carefully validated when possible, especially if they are to be interpreted at local scales'. Wolf and Ripple emphasized this warning by repeating it in the final section of their paper: ‘As mentioned in the limitations section, all protected areas and low footprint regions identified in our analysis must be thoroughly validated before any reintroduction attempts'. Nowhere in the paper did Wolf and Ripple state that any of the 130 sites are recommended for reintroduction. On the contrary, they acknowledged that ‘It is possible that for some of the large carnivore species, none of the candidate sites that we identified are appropriate for reintroductions'. However, Wolf & Ripple [1] does provide a useful roadmap for conservation planning via rewilding with large carnivores.

Wolf and Ripple's analysis relied heavily on current and historical species' geographical range maps. They noted that these maps have uncertainty and that the historical maps in particular provide only ‘rough approximations to species' historic ranges'. Given that such maps do not show fine-scale presence and absence information, it is unreasonable to expect high accuracy in their results. Miranda appears to have misunderstood the significance of Wolf and Ripple's results by not interpreting them in the context of the uncertainty inherent in species' geographical range maps. Ecology is an eclectic field that incorporates field experiments, observational studies, global analyses, mathematical modelling and other methodological approaches [22,23]. The diverse nature of techniques employed by ecologists reflects the idea that no single methodology is sufficient to address the important challenges and questions that lie ahead. As a macroecological study, Wolf and Ripple's analysis complements and builds upon decades of painstaking field research by providing a bird's-eye-view of large carnivore reintroduction possibilities. At the same time, the limitations and sources of error that Wolf and Ripple described in detail motivate further research to tackle the important questions of when and where reintroductions will have the greatest benefit and what steps are needed to ensure their long-term success.

## 5. Summary

The core findings of Wolf and Ripple are that there are many large protected areas and low human footprint regions where large carnivore species may have been extirpated and that some of these sites might have the potential for successful reintroductions, particularly when steps are taken to ensure adequate habitat, prey base and human tolerance. This work is important because large carnivore species' extensive range contractions and population declines mean that many of these species are now threatened and their ecological effects have been weakened globally [24]. Miranda did not address, let alone invalidate, the overarching significance of this analysis. Of course, global-scale models as in Wolf & Ripple [1] are just the first iteration in understanding where on the planet reintroductions might be most fruitful. A natural next step would be to get feedback from regional experts, and, if data are sufficient, model species distributions to make fine-scale predictions about optimal locations for rewilding (climatic and vegetation niches) [25]. A third step would be to rigorously establish (a) that the species is not in climatically suitable areas already (via detailed ground censuses) and (b) that the species is likely to have occurred in these areas historically. Finally, small-scale experimental reintroductions could be conducted to test whether conservation dollars are likely to be well spent. Recent work on condor reintroduction in the Pacific Northwest provides a concrete example of how fine-scale habitat modelling together with analysis of historical records and ancient DNA can be used to gain a more complete picture of reintroduction potential [26,27].

In summary, Miranda failed to provide any substantive criticism of Wolf and Ripple's use of scientific terms, selection of species to study or lists of reserves. Rather, he created his own ‘muddle' by introducing new terminology and making the vague suggestion that Wolf and Ripple should have analysed various other species. In doing so, we do not see how the comment by Miranda adds value to the discussion of how best to conserve large carnivores around the world. We see his comment as doing a disservice to the field of conservation, but we would welcome a more formal contribution by Miranda that extends (where possible) the research conducted by Wolf and Ripple to a broader range of species or improves species range maps through field surveys. This is what is really needed as opposed to unsubstantiated and unwarranted criticism.

## Data accessibility

This article has no additional data.
